# *Tulbaghia violacea* and *Allium ursinum* Extracts Exhibit Anti-Parasitic and Antimicrobial Activities

**DOI:** 10.3390/molecules23020313

**Published:** 2018-02-02

**Authors:** Sonja Krstin, Mansour Sobeh, Markus Santhosh Braun, Michael Wink

**Affiliations:** Institute of Pharmacy and Molecular Biotechnology, Heidelberg University, Im Neuenheimer Feld 364, 69120 Heidelberg, Germany; sobeh@uni-heidelberg.de (M.S.); m.braun@uni-heidelberg.de (M.S.B.)

**Keywords:** *Tulbaghia violacea*, *Allium ursinum*, garlic, sulfides, trypanocidal, leishmanicidal, antimicrobial activity, trypanothione reductase

## Abstract

Garlic has played an important role in culinary arts and remedies in the traditional medicine throughout human history. Parasitic infections represent a burden in the society of especially poor countries, causing more than 1 billion infections every year and leading to around one million deaths. In this study, we investigated the mode of anti-parasitic activity of “wild garlics” *Tulbaghia violacea* and *Allium ursinum* dichloromethane extracts against parasites *Trypanosoma brucei brucei* and *Leishmania tarentolae* with regard to their already known antimicrobial activity. We also evaluated their cytotoxic potential against human cells. Both extracts showed a relevant trypanocidal and leishmanicidal activity, although *L. tarentolae* was less sensitive. We determined that the probable mode of action of both extracts is the irreversible inhibition of the activity of *Trypanosoma brucei* trypanothione reductase enzyme. The extracts showed a mild cytotoxic activity against human keratinocytes. They also exhibited weak—in most cases comparable—antibacterial and antifungal activity. HPLC-MS/MS analysis showed that both extracts are abundant in sulfur compounds. Thus, for the first time, the ability of *Allium ursinum* and *Tulbaghia violacea* to kill *Trypanosoma* sp. and *Leishmania* sp. parasites, probably by binding to and inactivating sulfur-containing compounds essential for the survival of the parasite, is shown.

## 1. Introduction

Garlic has been used for thousands of years, predominantly for culinary purposes but also as remedy in traditional medicine. The ancient Israelis called garlic a “parasite-killer”; Hippocrates mentioned it as a medication against intestinal parasites [1]. Many studies have revealed the anticancer, antioxidant, antimicrobial, hypolipidemic, anti-inflammatory, and anti-parasitic effects of garlic [2,3,4,5,6,7].

*Tulbaghia violacea* Harv. (Amaryllidaceae) from Southern Africa is known as pink agapanthus or sweet garlic. *Tulbaghia* species have been utilised for culinary and ornamental purposes, but the genus is also medicinally relevant. Bulbs of pink agapanthus have been used in traditional medicine for treatment of pulmonary tuberculosis and against helminthes [8]. Studies have verified that extracts of different plant parts of *T. violacea* exhibit antibacterial, antifungal, anticancer, antioxidant and anthelmintic activities [9,10,11,12]. The European *Allium ursinum* L. (Amaryllidaceae) is also known as ramsons or bear’s garlic [13]. It has been included in the folk medicine as an antimicrobial agent, digestive and protective against cardiovascular diseases and respiratory problems. Recent research has confirmed the anticancer, anti-inflammatory, antiviral, antiplatelet, and hypolipidemic effects [5,14,15]. Both *T. violacea* and *A. ursinum* are commonly used edible species and are referred to as “wild garlics” [7,10,14].

**Figure 1:**
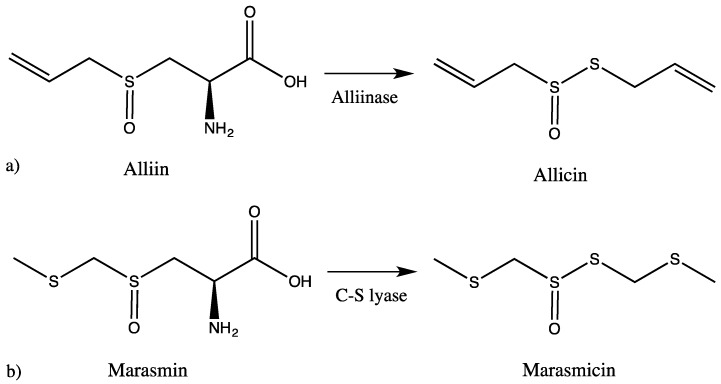
Pathways leading to the production of sulfur-containing compounds in (**a**) *Allium* and (**b**) *Tulbaghia*.

It is widely accepted that the distinct garlic-like odor and the specific taste derive from sulfur-containing secondary metabolites (SM), which are typical for both *Tulbaghia* and *Allium*. These sulfur compounds represent the majority of their chemical constituents, and it is strongly believed that they are the source of their pharmacological activity. The intact bulbs are odorless, but as soon as the plant tissue is damaged, an enzymatic reaction sets in, hydrolysing non-proteinogenic amino acids such as alliin or related SM, leading to volatile thiosulfinate products [10,14,16,17,18]. The chemical analysis of *Tulbaghia* has been rather neglected in comparison to *Allium* species. In *Allium*, when the bulbs are wounded, alliinase comes in contact with alliin and produces allicin, which in subsequent reactions is converted to other sulfur-containing compounds. In *Tulbaghia* species, the reaction starts instead of alliin from marasmin, which is enzymatically transformed to marasmicin, an analogue of allicin (Figure 1). This pathway is believed to be analogous to the alliinase pathway in *Allium* species [19].

Parasitic infections have always been a cause of mortality in human societies, especially in poor countries. More than 1 billion infections occur every year, which lead to around one million deaths. With only few registered drugs—often with severe side effects—against parasitic infections like trypanosomiasis and leishmaniasis, new therapeutic agents are urgently needed [20].

In parasites of the class Kinetoplastida, the enzyme vital for the redox system is trypanothione reductase, unlike humans in which glutathione reductase has the main role. Whereas glutathione is the main redox compound in vertebrates, it is trypanothione in Kinetoplastida. Although the two enzymes show similar characteristics, their disulfide specificity is different because the substrate binding site of trypanothione reductase is—unlike glutathione reductase—hydrophobic, wider, and negatively charged [21,22]. Their structural difference represents a basis for an interesting therapeutic opportunity: finding agents that have the ability to inactivate trypanothione reductase and trypanothione, but not glutathione reductase or glutathione.

Compared with other *Allium* species, such as garlic (*A. sativum*) and onion (*A. cepa*), the two species of wild garlic (*T. violacea* and *A. ursinum*) are not well characterized. Therefore, in this study, we investigated the phytochemistry of both species and the potential mode of anti-parasitic activity of dichloromethane bulb extracts against the parasites *Trypanosoma brucei brucei* and *Leishmania tarentolae*. For the first time, the trypanocidal and leishmanicidal activity of *T. violacea* and *A. ursinum* is reported, and evidence that the trypanothione reductase and trypanothione system is involved is provided. Antimicrobial activity was confirmed by testing seven Gram-positive and five Gram-negative bacteria (including several MDR strains) as well as two fungi.

## 2. Results and Discussion

Dichloromethane extracts from sweet garlic (*Tulbaghia violacea,* TV) and ramsons (*Allium ursinum,* AU) were evaluated for their anti-parasitic and antimicrobial activities, as well as for the potential molecular mode of anti-parasitic action.

**Figure 2:**
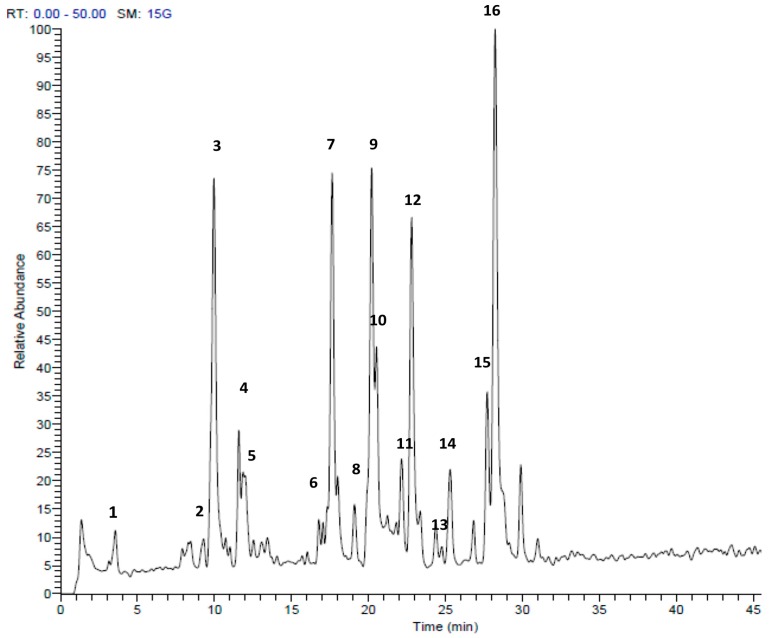
HPLC-MS/MS profile of *Allium ursinum* extract in the positive mode (+). Peak retention times correspond to compounds listed in Table 1.

**Figure 3:**
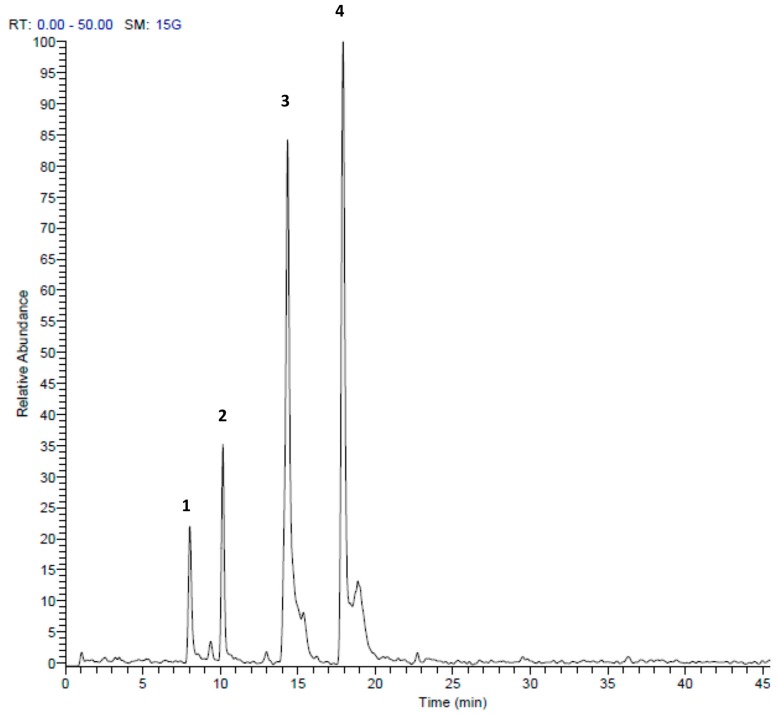
HPLC-MS/MS profile of *Tulbaghia violacea* extract in the positive mode (+). Peak retention times correspond to compounds listed in Table 2.

HPLC-MS/MS analysis clearly demonstrated that sulfur compounds are abundant in both extracts. We found the presence of allicin and ajoene in *A. ursinum* bulbs (Table 1, Figure 2). The TV extract also contained sulfur compounds, which differed from the AU extract. The main compound was marasmicin, which agrees with previous reports (Table 2, Figure 3) [19]. Compounds were identified according to retention time and MS data with reference to previous publications.

The extracts exhibited a weak and unspecific antibacterial and antifungal activity (Table 3). The AU extract was more effective against methicillin-resistant *Staphylococcus aureus* (MRSA) than TV with an MIC of 80 µg/mL. AU and TV extracts completely inhibited visible growth of *B. subtilis* at an MIC of 80 and 40 µg/mL, respectively. AU had moderate activity against *Pseudomonas aeruginosa* (MIC at 40 µg/mL). Both extracts inhibited the growth of *Candida* yeasts: 10 µg/mL AU were sufficient to not only inhibit growth, but kill *C. parapsilosis* at the same concentration as the positive control nystatin. The assay was used as a control to show that our results are in agreement with previous studies that reported weak antimicrobial activities of AU and TV extracts [29,30,31,32].

**Figure 4:**
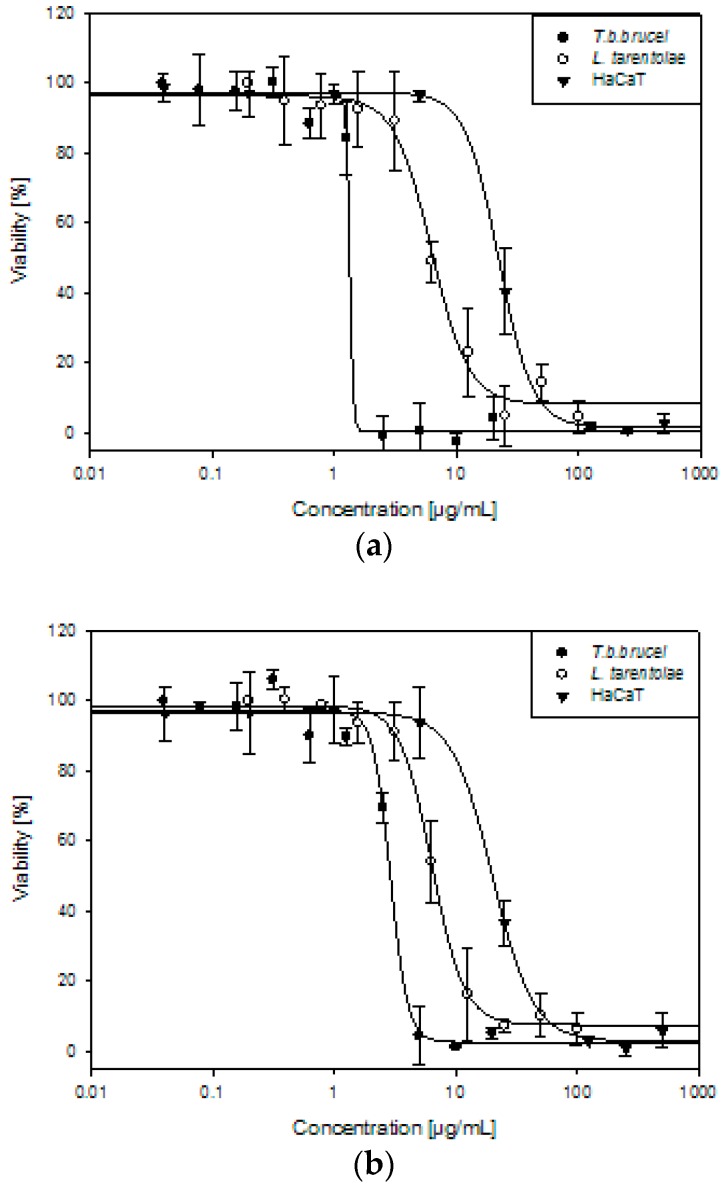
Trypanocidal, leishmanicidal, and cytotoxic effects of (**a**) *Allium ursinum* and (**b**) *Tulbaghia violacea* against *Trypanosoma brucei brucei* (*T. b. brucei*), *Leishmania tarentolae* (*L. tarentolae*), and HaCaT. Data illustrate the mean of three individual experiments.

AU and TV extracts killed 50% of *T.b.brucei* at 1.45 and 2.83 µg/mL, respectively. *L. tarentolae* parasites were less sensitive than trypanosomes, although still susceptible to the anti-parasitic activity of the two extracts. The corresponding dose-dependent curves are illustrated in Figure 4, while the IC_50_ values are documented in Table 4.

In order to compare the anti-parasitic effect of the AU and TV extracts with their cytotoxicity towards human cells and to evaluate their possible topical use against leishmaniasis, bacterial, and fungal infections, we used in vitro spontaneously transformed keratinocytes from histologically normal skin cells (HaCaT). The AU and TV extracts showed a moderate cytotoxic activity by killing 50% of the cells at concentrations of 23.71 and 21.35 µg/mL, respectively. The selectivity indices (SI), with values of 16 and 8 for AU and TV in trypanosomes, respectively, are more favorable when comparing cytotoxic activity towards HaCaT cells with the trypanocidal then with leishmanicidal effect. AU could be potentially used topically in the first phase of *T. brucei* infection, in which parasites can be found in the skin after the tsetse fly bite for up to a few days. The SI and IC_50_ values are documented in Table 4.

**Figure 5:**
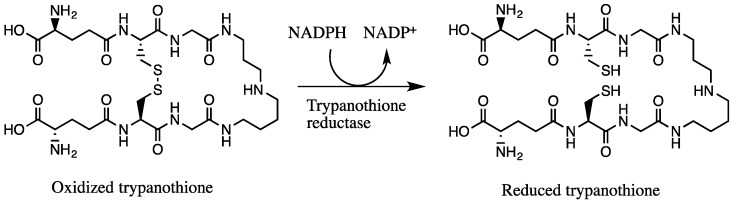
Reduction of oxidized form of trypanothione by trypanothione reductase with NADPH.

**Figure 6:**
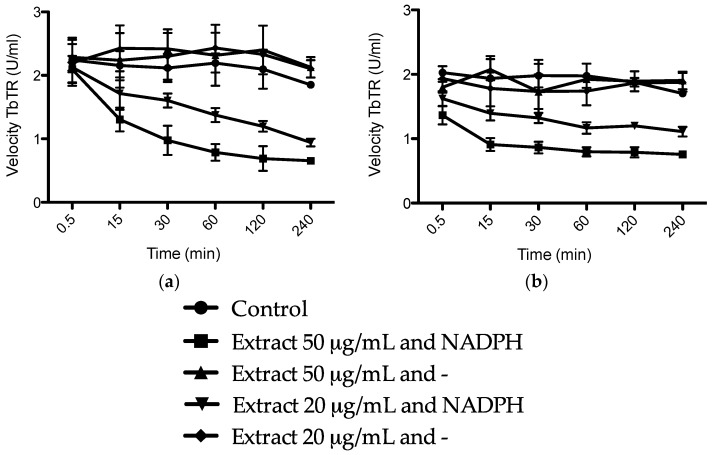
Time course of irreversible inhibition of *Trypanosoma brucei* trypanothione reductase (TbTR) by 50 and 20 μg/mL of (**a**) *Allium ursinum* and (**b**) *Tulbaghia violacea* extracts. Data are shown as mean of three independent experiments ± SD.

We hypothesized that the trypanocidal and leishmanicidal effect is caused by sulfur compounds being able to establish disulfide (–S–S–) bonds with free thiol (–SH) groups, thus inactivating them. Free thiol groups are present inside the parasite at the active sites of some of the vital substances for the survival of trypanosomes, like the NADPH flavoenzyme trypanothione reductase and trypanothione itself, which provide an intracellular reducing environment and play a major role in the defense of oxidative stress (Figure 5) [22,33]. The trypanothione redox system is unique to Trypanosomatida, while humans rely on the glutathione reductase/glutathione system. In order to evaluate our hypothesis, we investigated the inhibition of the enzyme TbTR by the extracts, and the results are illustrated in Figure 6. In our experiment, we confirmed that both extracts are irreversible inhibitors of the TbTR enzyme: 50 µg/mL of AU and TV extract inhibited the enzymatic activity of the TbTR by 71% and 63% within 4 h, respectively. One of the compounds we found in the AU extract is ajoene. Our data on TbTR inhibition are in agreement with a previous study [34]; they provided evidence that ajoene is an inhibitor of trypanothione reductase. TV seems to inhibit the TbTR in a rapid manner; after 0.5 min, a decrease in the TbTR activity was noticeable. It could be that marasmicin, the main compound in the TV extract, is responsible. In this study, the ability of an extract containing marasmicin to inhibit a vital enzyme in the survival of Kinetoplastida and kill parasites efficiently is shown for the first time. In vivo studies are necessary to confirm the anti-parasitic potential of marasmicin in a living organism in order to break new ground for a possible development of a new drug.

Thus, for the first time, evidence of the relevant anti-parasitic activity of *Allium ursinum* and *Tulbaghia violacea* against *Trypanosoma* and *Leishmania* parasites is provided. Future studies in which the mechanism of action and the activity in vivo are analyzed in depth are warranted. Our results indicate a probable mechanism of action: sulfur compounds inhibit compounds vital for the survival of parasites, such as trypanothione reductase. It should be noted, however, that both trypanosomatid parasites used for the assays are not pathogenic for humans. Further studies with clinically relevant species/strains have yet to be conducted.

## 3. Materials and Methods

### 3.1. Chemicals

Hemin chloride was purchased from Merck Millipore (Darmstadt, Germany). Medium DMEM with Glutamax, MEM, non-essential amino acids (NEAA), penicillin, streptomycin, trypsin-EDTA, and l-glutamine came from Gibco^®^ Invitrogen (Darmstadt, Germany). Doxorubicin hydrochloride was acquired from the Heidelberg University Hospital. Nystatin and ampicillin were bought from AppliChem (Darmstadt, Germany). The rest of the chemicals was obtained from Sigma-Aldrich GmbH (Steinheim, Germany).

### 3.2. Extract Preparation

Bulbs from *Allium ursinum* and *Tulbaghia violacea* were acquired from the Botanical Garden of Heidelberg University. To prepare the extracts, 30 g of bulbs from each species were crushed and homogenized in a commercial blender, soaked in 100 mL of dichloromethane (CH_2_Cl_2_) and left on a stirrer overnight. Extracts were then filtered using filter paper and excess water was removed from the dichloromethane phase with anhydrous sodium sulfate. The dichloromethane extracts were concentrated using a rotary evaporator at 40 °C. Extracts were kept at −80 °C until further analysis.

### 3.3. Cell Lines

For the experiments, cell lines of *Trypanosoma* and *Leishmania* were used which are not infectious for humans. Our *T. b. brucei* blood-stream cell line was originally obtained from Prof. Peter Overath (Max-Planck-Institut für Biologie, Tübingen, Germany). It was maintained in complete Baltz medium [35]. Immortalized human keratinocytes (HaCaT; in collaboration with Prof. Stefan Wölfl, Institute for Pharmacy and Molecular Biotechnology, Heidelberg, Germany) were grown in Dulbecco’s modified Eagle’s medium (DMEM) with Glutamax supplemented with 10% fetal calf serum (FBS), 1% of non-essential amino acids (NEAA), 100 U/mL penicillin, and 100 µg/mL streptomycin. Both cell lines were cultivated at 37 °C, 5% CO_2_, and 95% humidity. *Leishmania tarentolae,* originally isolated from geckos [36], was kindly provided by Prof. Marcel Deponte (Zentrum für Infektiologie, Parasitologie Universitätsklinikum Heidelberg, Heidelberg, Germany). Parasites in the promastigote stage were cultured at 26 °C in BHI supplemented with hemin chloride (10 μg/mL), 100 U/mL penicillin, and 100 µg/mL streptomycin. All experiments were performed with cells being in their logarithmic growth phase.

### 3.4. Screening of Trypanocidal, Leishmanicidal and Cytotoxic Activities

The trypanocidal, leishmanicidal, and cytotoxic activities of the extracts were investigated using the MTT cytotoxicity assay [37]. The MTT assay is based on the viability of the cells being measured by the reduction in tetrazolium salt (3-(4,5-dimethylthiazol-2-yl)-2,5-diphenyltetrazolium bromide; MTT) in the mitochondria of living cells to its colored formazan salt. Serial dilutions of extracts were obtained in the medium corresponding to the cell line, in which the maximal concentration of solvents did not exceed 2%. Incubation of 2 × 10^4^ *T. b. brucei* or 20 × 10^4^ *L. tarentolae* cells/well took place with extracts in a serial two-fold dilution for 48 h in a 96-well plate (Greiner Labortechnik, Frickenhausen, Germany). Afterwards, 0.5 mg/mL of MTT was added. After 4 h (*T. b. brucei*) and 90 min (*L. tarentolae*) incubation, the formazan crystals that were produced by viable cells were dissolved in 100 µL of DMSO, and the plates were shaken at room temperature for 10 min. The plates were then read at 570 nm using a BiochromAsys microplate reader (Biochrom, Cambridge, UK). After 2 × 10^4^ HaCaT cells/well were seeded and incubated for 24 h, the cells were then incubated with a serial dilution of the extracts for 24 h. The MTT assay was then carried out as previously stated. The trypanocidal drug suramin, the leishmanicidal amphotericin B, and the cytotoxic doxorubicin were used as positive controls. Cell viability was additionally evaluated by light microscopy. Using the four parameter logistic regression (SigmaPlot^®^ 11.0, San Jose, CA, USA), a sigmoidal curve was fitted, and the IC_50_, which represents a 50% reduction in viability compared to non-treated cells, was calculated.

### 3.5. Selectivity Index

Cytotoxicity against *T. b. brucei* and *L. tarentolae* was compared to that against HaCaT cells, in order to calculate the selectivity index (SI). SI represents the ratio of the IC_50_ value for mammalian cells divided by the IC_50_ in trypanosomes or leishmanial parasites. Higher values of SI mean selectivity activity against parasites and are therefore important if a utilization of a chemotherapeutic agent is considered.

### 3.6. Trypanosoma brucei Trypanothione Reductase (TbTR) Inhibition Assay

The method was originally developed by Jockers-Scherubl et al. (1989) [38]. Recombinant TR and trypanothione were synthesized following previously published procedures [39,40]. In a 200 μL reaction mixture, 20 and 50 μg/mL of extracts or equal amount of the solvent were incubated with 25 μL of TbTR (20 U/mL) in the presence and absence of 400 μM NADPH at room temperature for 240 min. After 0.5, 15, 30, 60, 120, and 240 min, a 5 μL aliquot was removed and used to determine the remaining activity of the enzyme in a standard assay [41]. The third mixture contained buffer, TbTR, the extract, and no NADPH to check if the oxidized enzyme can react irreversibly.

### 3.7. Antimicrobial Tests

Extracts were tested against seven Gram-positive and five Gram-negative bacteria as well as two fungi at concentrations between 320 and 2.5 µg/mL. Gram-positive microorganisms were *Bacillus subtilis* ATCC 6051, methicillin-resistant *Staphylococcus aureus* (MRSA) NCTC 10442, the clinical isolate MRSA KL 21790, *Staphylococcus epidermidis* ATCC 14990, *Enterococcus faecalis* ATCC 29212, vancomycin-resistant VanB positive *Enterococcus faecalis* (VRE) ATCC 51299, and *Streptococcus pyogenes* ATCC 12344. Gram-negative bacteria were *Escherichia coli* ATCC 25922, enterohemorrhagic *Escherichia coli* O157:H7 ATCC 35150, *Klebsiella pneumoniae* ATCC 700603, the clinical isolate *Klebsiella pneumoniae* UL 30082, and *Pseudomonas aeruginosa* ATCC 27853. Anti-candida activity was determined using *Candida albicans* ATCC 90028 and *Candida parapsilosis* ATCC 22019. All organisms were courtesy of the Department of Infectious Diseases, Medical Microbiology and Hygiene, Heidelberg University, Heidelberg, Germany.

Minimum inhibitory concentration (MIC) was obtained by means of broth microdilution following the method of CLSI [42]. Bacteria were maintained on Columbia Agar supplemented with 5% sheep blood and fungi on Sabouraud Dextrose agar (SDA). Liquid medium used for the determination of MICs was Müller–Hinton broth for bacteria except for *Streptococcus* spp. and *Enterococcus* spp., which were tested in Brain–Heart Infusion broth (BHI). Fungal liquid medium was RPMI 1640. For the determination of the minimum microbicidal concentrations (MMCs), defined volumes of each well with concentrations ≥ MIC were streaked on agar plate and incubated for 24 h (bacteria) and 48 h (fungi). The minimum concentration of the extracts killing at least 99.9% of the initial inoculum was regarded as MMC. Solvent growth and sterility controls were included in the tests. Ciprofloxacin and ampicillin served as positive control for bacteria, nystatin was used for fungi. The tests were conducted in duplicate per plate and performed thrice.

### 3.8. HPLC-MS/MS Analysis

High performance liquid chromatography–mass spectrometry (HPLC-PDA-MS/MS) was used to identify the chemical composition of the extracts. The LC system was Thermofinnigan (Thermo Electron Corporation, Waltham, MA, USA) coupled with an LCQDECA XP Plus ion trap mass spectrometer with an ESI source. The separation was achieved using a C18 reversed-phase column (Zorbax Eclipse XDB-C18, Rapid resolution, 4.6 × 150 mm, 3.5 µm, Agilent, Santa Clara, CA, USA). A gradient of water and acetonitrile (0.1% formic acid each) from 5% to 70% ACN in 45 min was applied and then kept for 5 min at the last conditions. The flow rate was 1 mL/min and a splitter was used to deliver only 50% of the sample into the analyzer. The samples were injected automatically using autosampler surveyor ThermoQuest. The instrument was controlled by Xcalibur software to collect the UV chromatogram using PDA mode and the MS data. The MS operated in the positive mode with a capillary voltage of −10 V, a source temperature of 275 °C, and high purity nitrogen as a sheath and auxiliary gas at a flow rate of 80 and 40 (arbitrary units), respectively. The ions were detected in a full scan mass range of 50–2000 *m*/*z* [43].

## Reference

## Figures and Tables

**Table 1:** Identification of secondary metabolites in *Allium ursinum* extract by LC-ESI-MS/MS.

Peak No.	t_R_	[M + H]^+^	Area %	Proposed Compound	Reference
1	3.56	151	1.67	Methyl pentyl disulfide	[23]
2	9.73	137	0.90	Methanesulfinothioic acid S-(*E*)-1-propenyl ester	[24]
3	9.93	137	14.02	Methanesulfinothioic acid S-(*Z*)-1-propenyl ester	[24]
4	11.57	137	2.96	S-Methyl 1-propenesulfinothioate	[24]
5	11.83	137	3.75	S-1-Propenyl methanesulfinothioate	[24]
6	16.77	185	1.07	Methyl 1-(methylsulfinyl)propyl disulfide	[25]
7	17.65	163	12.83	Allicin	[26]
8	19.92	209	1.67	(*E*)-1-allyl-2-(3-(methylesulfinyl)prop-1-en-1-yl)disulfane	Tentative
9	20.20	163	12.39	2-Propene-1-sulfinothioic acid S-(*E*)-1-propenyl ester	[24]
10	20.54	163	5.78	Propene-1-sulfinothioic acid S-(*Z*)-1-propenyl ester	[24]
11	22.16	211	2.45	1-(methylsulfinyl)propyl(*E*,*Z*)-1-propenyl disulfide	[25]
12	22.81	211	10.88	Methyl 1-(2-propenylsulfinyl)propyl disulfide	[25]
13	23.37	211	1.22	1-(methysulfinyl) propyl 2-propenyl disulfide	[25]
14	25.31	235	3.27	Ajoene	[26]
15	27.69	237	4.65	(*E*)-1-propenyl 1-(1-propenylsulfinyl)propyl disulfide	[25]
16	28.25	237	20.50	2-Propenyl 1(2-pro-penylsulfinyl) propyl disulfide	[25]

**Table 2:** Identification of secondary metabolites in the *Tulbaghia violacea* extract by LC-ESI-MS/MS.

Peak No.	t_R_	[M + H]^+^	Area %	Proposed Compound	Reference
1	8.01	157	4.21	S-Propyl thiosulfate	Tentative
2	10.16	303	11.30	Di-(1-S-sulfoxymethyl-butyl)-disulfide	[27]
3	14.34	203	32.03	S-(2-Pyrrolyl)cysteine S-oxide	[28]
4	17.92	203	52.46	2,4,5,7-tetrathiaoctane4-oxide (marasmicin)	[19]

**Table 3:** Antimicrobial activity of *Allium ursinum* (*A. ursinum*) and *Tulbaghia violacea* (*T. violacea*) extracts against different G-positive and G-negative bacteria and *Candida* yeasts. MIC (minimum inhibitory concentration) and MMC (minimum microbicidal concentration) values are shown as µg/mL. Positive controls are ciprofloxacin, ampicillin, and nystatin.

Gram Type	Sample Indicator Strain	*A. ursinum*		*T. violacea*		Ciprofloxacin	Ampicillin	Nystatin
		MIC	MMC	MIC	MMC	MIC	MIC	MIC
+	*Bacillus subtilis*	80	160	40	80	≤0.03	≤0.03	NT
+	MRSA	80	>320	320	>320	0.03	16	NT
+	MRSA CI	80	>320	320	>320	4	16	NT
+	*Staphylococcus epidermidis*	80	>320	160	>320	0.03	0.5	NT
+	*Enterococcus faecalis*	320	>320	>320	>320	0.5	1	NT
+	VRE	>320	>320	>320	>320	0.5	1	NT
+	*Streptococcus pyogenes*	160	160	160	160	0.13	<0.03	NT
−	*Escherichia coli*	80	>320	80	320	≤0.03	4	NT
−	*Escherichia coli* EHEC	160	>320	160	>320	≤0.03	4	NT
−	*Klebsiella pneumoniae*	80	>320	160	>320	0.125	>64	NT
−	*Klebsiella pneumoniae* CI	160	>320	160	>320	<0.03	32	NT
−	*Pseudomonas aeruginosa*	40	>320	160	>320	≤0.03	>64	NT
F	*Candida albicans*	20	20	20	20	NT	NT	10
F	*Candida parapsilosis*	10	10	20	40	NT	NT	10

NT: not tested; CI: clinical isolate; F: fungus.

**Table 4:** Trypanocidal, leishmanicidal, and cytotoxic activity of *Allium ursinum* and *Tulbaghia violacea* extracts against *Trypanosoma brucei brucei* (*T. b. brucei*), *Leishmania tarentolae* (*L. tarentolae*), and HaCaT cells. The values are expressed as mean IC_50_ (µg/mL) ± SD; nt: not tested; SI: selectivity index.

	*T. b. brucei*	*L. tarentolae*	HaCaT	SI	
				HaCaT/*T. b. b.*	HaCaT/*L. t.*
*Allium ursinum*	1.45 ± 0.14	5.87 ± 0.48	23.71 ± 2.66	16	4
*Tulbaghia violacea*	2.83 ± 0.23	6.29 ± 0.58	21.35 ± 2.54	8	3
Suramin	0.13 ± 0.01	nt	nt	-	-
Amphotericin B	nt	0.13 ± 0.02	nt	-	-
Doxorubicin	nt	nt	1.04 ± 0.35	-	-
